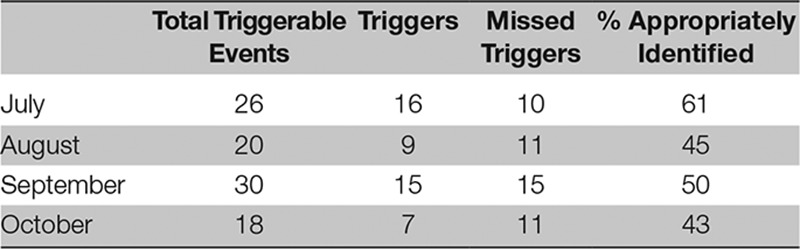# Recognition of Sepsis Triggers in a Mixed EMR Community Hospital

**DOI:** 10.1097/pq9.0000000000000071

**Published:** 2018-04-17

**Authors:** Michael D. Salt, Adam Beaton, Krista Schreiber, Bernardita Gamallo

**Affiliations:** From the * Goryeb Children’s Hospital, Morristown, N.J.; † Pediatric Intensive Care Unit, Goryeb Children’s Hospital, Morristown, N.J.

## Abstract

**Background:**

Early recognition of sepsis and rapid intervention has been proven to decrease both morbidity and mortality. However, early recognition continues to be a problem across all pediatric settings including our hospital.

**Objectives:**

Having no capable EMR system, our institution has implemented a manual trigger tool (Fig. 1) to help earlier identify those at risk and prevent progression to severe sepsis.

**Methods:**

All patients admitted to the pediatric floor at Goryeb Children’s Hospital, a community hospital, with 1,900 inpatient admissions annually, were monitored for abnormal vitals meeting criteria as defined by our trigger tool. With our key driver being prevention, we instituted a manual trigger tool as the secondary driver to help us achieve our goal. We retrospectively collected data on the number of patients who were identified and documented versus those who met criteria that were not documented. After the first month of data collection, further sepsis education was provided to the resident house staff and nursing staff. Additionally, vital sign criteria for our trigger were placed on all monitors (Fig. 2).

**Results:**

Over the first 4 months, from July to October, after instituting our manual trigger tool, 55%, 45%, 50%, and 43% of vital signs were appropriately identified and documented (Table 1).

**Conclusions:**

Our ability to recognize abnormal vitals in a potentially septic patient continues to be below our goal of 80% at 6 months with a manual trigger process. Areas for possible improvement include further education to the staff and integration of EMR with automated trigger tools.

**Fig. 1.**
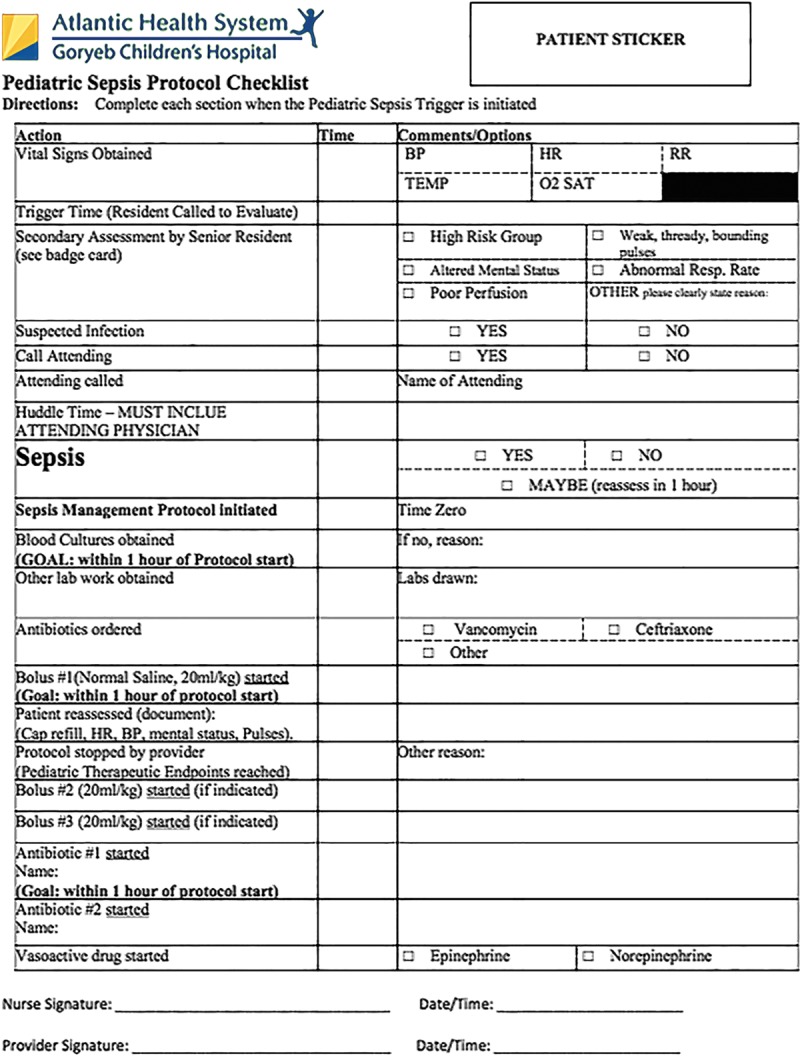
Upon identification as meeting trigger criteria, pediatric residents and nursing staff are required to fill out the above form indicating reason for trigger, initial assessment, interventions undertaken, and reevaluations.

**Fig. 2.**
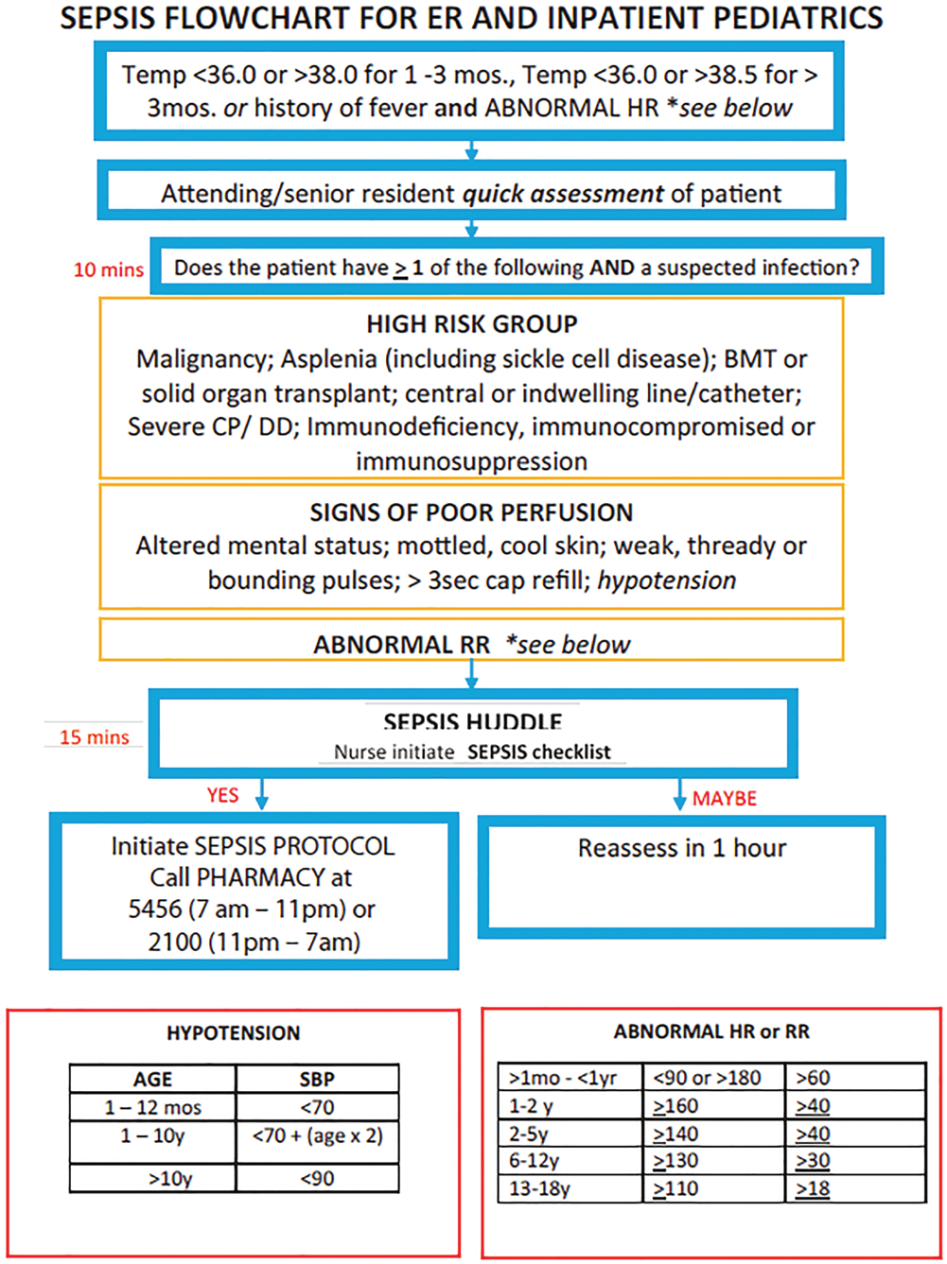
Pediatric sepsis trigger cards are carried by all pediatric residents to ensure timely response to abnormal vitals. These cards are now also permanently placed on all portable vital machines and available for all nursing staff and medical technicians responsible for obtaining vitals.

**TABLE 1.**